# Parenting experiences of Chinese mothers living with a mental illness

**DOI:** 10.1186/s12888-021-03581-9

**Published:** 2021-11-23

**Authors:** Lingling Chen, Kitty Vivekananda, Lili Guan, Andrea Reupert

**Affiliations:** 1 Faculty of Education, Monash University, 19 Ancora Imparo Way, Clayton, VIC 3800 Australia; 2 Peking University Sixth Hospital, Peking University Institute of Mental Health, NHC Key Laboratory of Mental Health (Peking University), National Clinical Research Centre for Mental Disorders (Peking University Sixth Hospital), Beijing, China

**Keywords:** Chinese mothers, Mental illness, Parenting, Qualitative, IPA

## Abstract

**Background:**

Although the experiences of mothers with mental illness are well researched in Western countries, little is known about the experiences of Chinese mothers. This study aims to explore the experiences of family life and parenting of Chinese mothers, in the context of their mental illness.

**Methods:**

Fourteen Chinese mothers with mental illness undertook in-depth, semi-structured interviews. Interpretative Phenomenological Analysis was employed to guide the data analysis.

**Results:**

Seven themes were identified: motherhood as a central identity, the stigma associated with being a mother with mental illness, participants’ perceptions about the impact of mental illness on parenting and their children, experiences of talking to children about mental illness, how having children impacts mothers’ illness and recovery, and support obtained and needed. Similar to Western mothers, Chinese mothers experienced stigma and fluctuating mental illness symptoms which impacted on parenting. Unlike mothers based in Western countries, the mothers interviewed in this study highlighted complicated co-caring relationships with parents-in-law and did not raise child custody concerns.

**Conclusions:**

Mental health professionals need to have the skills to identify and recognize the mothering role of their clients. Culturally sensitive interventions are required to assist Chinese families where mothers have a mental illness. Future research is required to investigate family experiences of parental mental illness from the perspectives of children, partners, and mental health professionals.

**Supplementary Information:**

The online version contains supplementary material available at 10.1186/s12888-021-03581-9.

## Background

Mental illness can impact parenting in various ways which in turn may potentially negatively impact children [1, 2]. However, not all children will be adversely impacted as multiple moderating factors mediate children’s outcomes, such as the quality and quantity of support available to the family and the severity and chronicity of the parent’s illness [3, 4]. Much of the research in this area has focused on the needs of mothers from Western countries, who experience various mental health challenges.

The research collected to date from countries such as Australia, the United States (US), and the United Kingdom (U.K.), has found that mothers, who have a mental illness, consider motherhood as a rewarding and important role in their lives [5, 6]. Having or caring for children brings these mothers purpose in life and positive emotions [7, 8], provides them with opportunities to connect with communities [7], and protects them from engaging in high-risk behaviours [9]. Parenting can also play a key role in one’s recovery, especially if parenting responsibilities are embedded into treatment plans [10].

Simultaneously, mothering and managing a mental illness can be challenging [11, 12]. As a result, mothers may become overly permissive, harsh, and inconsistent when disciplining children [8, 13, 14]. Some mothers are concerned about the impact of their illness on children [5, 15], and as a result, experience profound feelings of guilt, regret and sadness [6, 16]. In some families, role reversal occurs, whereby children assume the caregiving responsibilities for their parents and/or siblings [17, 18]. Stigma and the fear of losing child custody can be significant barriers for some mothers seeking help with their parenting [5].

The experiences of Chinese mothers with mental illness may differ from those of Western mothers. Traditional and gendered expectations and norms of motherhood based on Confucian ethics of filial piety and harmony are particular to Chinese parenting [19]. In traditional Confucian societies, mothers are assumed to be the primary caregivers, and so may feel responsible or are blamed for family issues such as juvenile delinquency or intergenerational conflict [20, 21]. As balancing the demands of parenting and their mental illness is an important experience for Western mothers [6], it is meaningful to explore how social expectations of motherhood may impact the parenting experiences of Chinese mothers with a mental illness. In addition, the previous one-child policy [22] changed the Chinese family structure, resulting in a “4–2-1” phenomenon whereby six caregivers, comprising two parents and four grandparents, pool all their resources to raise the only child [22]. This arrangement can be supportive in managing childcare and housework, but may increase family conflicts around childrearing [22]. Similarly, some studies conducted with Chinese mothers with postpartum depression (PPD) have found that family members, especially husbands and parents-in-law, may at times support, and at other times, burden mothers [23, 24]. How this relates to other mothers with different mental illness diagnoses is not clear. Overall, cultural differences around parenting and family life between China and Western countries [25, 26] highlight the need to explore the parenting experiences of Chinese mothers, and their needs and preferences for support.

A recent systematic review [19] identified nine, qualitative papers describing the experiences of mothers, who have a mental illness, living in mainland China, Hong Kong, Macao, and Taiwan. Six papers included mothers with PPD while three focused on mothers with any serious mental illness (SMI). Similar to mothers from Western countries, the review found that Chinese mothers faced the challenges of managing their mental illness while simultaneously assuming parenting responsibilities. The review pointed out key differences between the experiences of Western and Chinese mothers in regard to the influence of parents-in-law, alongside a lack of formal professional support in their parenting role [15, 19]. Only three studies in this review were conducted in mainland China and these all focused on mothers with PPD [24, 27, 28]. As some research suggests that the experiences of mothers with PPD are different from those with other mental health diagnoses [4], there is a need to investigate Chinese mothers with mental illness diagnoses other than PPD. Moreover, only three studies were considered methodologically rigorous, with common problems around researcher reflexivity and a lack of transparency in the analytical process [19]. In conclusion, the lack of high-quality qualitative research on Chinese mothers who have a mental illness is limited and a broader focus on mothers with mental health diagnoses other than PPD is required.

There is a need for exploratory phenomenological research to investigate the parenting experiences of Chinese mothers with diverse mental health diagnoses. The purpose of this paper was to explore the parenting experiences and needs of Chinese mothers with mental illness. The research questions include i) How do Chinese mothers who have a mental illness experience parenting? ii) How do Chinese mothers perceive the support they have received? What are their needs? Such information can be used to inform the development of culturally appropriate services and interventions.

## Methods

A qualitative approach was employed, using Interpretative Phenomenological Analysis (IPA) as the framework to guide data analysis [29]. IPA is concerned with understanding personal lived experiences through a process of “double hermeneutic” in which “the researcher is trying to make sense of the participant trying to make sense of what is happening to them” ([30] p. 10). It is widely used in health psychology [30] and allows for a focus on a small number of cases. Four to ten cases are considered appropriate [29] given the focus on examining in detail the convergence and divergence of participants’ lived experiences.

### Participants and recruitment

Eligibility for inclusion were women with a current or past experience of mental illness, living in mainland China and having at least one child under 18 (either living with them or not). They also needed to be in treatment if they were currently ill, able to provide informed consent, and present coherently during the interview. Ethics approval for this study was provided by the university human research ethics committee (project ID: 21499). Forty-three female applicants expressed an initial interest to participate in the study during the recruitment through social media advertising (i.e., WeChat) and online peer support groups between June and September 2020. Fourteen participants with various mental health diagnoses met the inclusion criteria and provided verbal and electronic informed consent. Participants’ demographics and self-reported mental illness diagnosis are presented in Tables 1 and 2 respectively.

**Table 1:** Participant demographics (*n* = 14)

Demographics	Number of participants (n = 14)
Age range	
20–29 years	1
30–39 years	8
40–49 years	5
Marital status	
Currently married to children’s father	12
Divorced from children’s father, no new relationship	1
Divorced from children’s father, new relationship	1
Education	
Middle school	1
High school/ Technical secondary school	1
College/ University	8
Postgraduate	3
Employment status	
Full-time job	11
Part-time job	1
Freelancers	1
Housewife	1
Living status during the illness	
Living with extended family	8
Not living with extended family	6
Age of children at the time of interview	
1–6	5
7–12	10
13–18	4
Number of children	
1	9
2	5
Monthly household income (CNY)	
6750-8250	1
8250-9750	1
9240–12,040^a^	2
9750-11,250	1
11,250-12,750	2
12,750-14,250	1
More than 14,250	5
Not disclosed	1

^a^This indicator refers to the monthly household income of two participants from rural areas; the other indicators are for participants from urban areas

Calculated from data provided by the National Bureau of Statistics of China (2012, 2019), the average monthly household income in urban and rural areas is CNY 5295 and CNY 3738 respectively

**Table 2:** Self-reported mental illness diagnosis (*n* = 14)

Variable	Number of participants (***n*** = 14)
Diagnosis	
Anxiety	2
Major depression	4
Postnatal depression	3
Anxiety and depression	1
Schizophrenia	1
Bipolar disorder	3
State of mental illness	
In treatment	7
Recovered from symptoms	6
Neither in treatment nor recovered from symptoms ^a^	1
Source of diagnosis	
Reported that a psychiatrist provided diagnosis	12
Self-diagnosed	2
First diagnosis	
Prior to first birth	3
After giving birth	11
Time duration since the first diagnosis	
Less than 1 year	3
1–5 years	8
6–10 years	1
11–20 years	0
More than 20 years	2

^a^Although this mother was not in treatment, the interviewer ensured that she was able to provide informed consent. She also presented coherently during the interview

### Data collection

A semi-structured interview schedule based on previous literature and the research questions was employed (refer to Additional file 1). The schedule included questions on mothers’ perspectives about their parenting role, their experiences of family life in the context of their illness, and mothers’ support system (or lack thereof). Twelve interviews were conducted over the telephone and two were conducted using video calls. All were conducted by the first author in Mandarin from June to September 2020. Rapport was established and informed consent was obtained during a pre-interview call. The average interview time was 171 min (Range = 64–294 min), with two participants interviewed in two sessions. All interviews were audio-recorded, except for one who refused to be recorded but allowed the interviewer to take notes. Memos were taken during and immediately after each interview. Member checking was conducted to correct any possible inaccuracies or identifiable information, with eight participants clarifying their transcripts or providing additional information.

### Analysis

Verbatim transcripts in Chinese were coded line-by-line and analysed by the first author using IPA [29]. NVivo 12 was used to manage, index, and retrieve data. Representative excerpts are presented below tagged with participants’ pseudonyms. To assure the trustworthiness of the coding process and translation, codes and translation were firstly undertaken by the first author and reviewed by the other Chinese-speaking researcher for two cases given time constraints and the complexity of the translation process. The translations were then checked for readability and comprehensibility by the two researchers who were native English speakers. Comprehensive discussions were then pursued, whereby codes in the English translation and theme structures were discussed before final inclusion into this paper.

At the time of this research, the first author was an international student and a bilingual Mandarin-English speaker with a background in psychology, completing a Ph.D. She has previous research and work experience with Chinese families and is an adult child of a mother with mental illness. The research team brought expertise in qualitative approaches, and research and clinical experiences of Australian and Chinese families. Throughout this study, the first author engaged in self-reflection through journaling [31], as well as debriefing and discussion with the supervision team to reduce the potential impact of personal bias on this study.

## Results

Seven interrelated themes with nine sub-themes were identified (see Table 3). Overall, motherhood was identified as a central identity by all participants even though they experienced much stigma from others about caring for children while managing a mental illness. Nonetheless, having a mental illness impacted participants’ parenting role and behaviours, which in turn resulted in concerns about how their illness impacted on children. Accordingly, they adopted multiple strategies to reduce this impact, including talking to their children about their mental illness. Not only did their illness impact parenting and children, but having children impacted participants’ illness and recovery. Though there was some social support for participants in their parenting role, a considerable need for more effective parenting support was identified. These themes are detailed further below.

**Table 3:** Schematic of themes

Themes	Sub-themes
1 Motherhood as a central identity	
2 The stigma associated with being a mother with mental illness	
3 The impact of mental illness on parenting	• Impact of fluctuating moods on parenting • Feeling guilty, overwhelmed, and helpless • Self-acceptance
4 Perceptions about the impact of the mental illness on children	• Hereditary worries • Impact on children’s development • Reducing the negative impact of the mental illness on children
5 Experiences of talking to children about mental illness	
6 How having children impacts mothers’ mental illness and their recovery	
7 Support obtained and needed	• Helpful support • Unhelpful support or a lack of support • Support needed

### Motherhood as a central identity

All participants valued motherhood as a central identity in their lives, for example, “My other jobs are just phases, but the job as a mother is my lifelong career” (M03). One participant who was not in paid employment noted, “I still want to make a difference. ... Since I don’t have a company to contribute to, I’ll contribute to my son” (M06). Another believed that motherhood brought her a sense of completeness, “I am a daughter, a wife ... and a company employee ... I have experienced the process of motherhood. All these brought me a complete life experience” (M14).

### The stigma associated with being a mother with mental illness

Six participants described different types of the stigma associated with being a mother with mental illness. One participant with schizophrenia described experiencing public stigma, with a relative accusing her of being “neither a good mother nor a good wife” (M13). Self-stigma was experienced, “I saw other mothers having such a good relationship with their children … I felt like I had lived in vain” (M04). Likewise, another felt “a sense of shame” because “other parents had a lot in common to talk about … [but] I was dumb and I didn’t talk” (M13).

Two participants feared their children might experience stigma because of their mental illness. One reported her “son was excluded” (M13) from birthday parties because of her illness while another kept “all my information blocked up” because “I don’t know if I’ll be the cause for my daughter being teased … or … bullied at school” (M03).

### The impact of mental illness on parenting

This theme focuses on participants’ perceptions regarding how their illness impacted parenting with three subthemes identified: i) the impact of fluctuating moods on parenting; ii) feeling guilty, overwhelmed, and helpless; and iii) self-acceptance.

#### Impact of fluctuating moods on parenting

During well times, many participants (*n* = 10) reported being “rational” (M09), “tolerant” (M13), “patient” (M02), and “energetic” (M12) with their children. They also engaged in energy-demanding outdoor activities with children (M09 & M14). Conversely, when participants were depressed, anxious, or feeling unwell, many (*n* = 9) described reduced involvement in caregiving and communication due to lacking “motivation” (M04), “energy” (M12), and because of rumination (M06). Discipline challenges with teenagers were experienced (M06, M09, M10, M13) and where M09 reported getting “angry very easily” with her adolescent daughter.

There were specific issues associated with psychosis where one participant reported making “my son think he was the top of the universe” and beating her son to “transmit … masculine energy” (M06). This participant also reported having ideas of killing both herself and her son during psychotic episodes and being hospitalized without informing her little child. Another participant could not remember what happened during a psychotic episode but was told by her mother that she “threw her [child’s] phone into the toilet [and] ... hit her” (M13).

#### Feeling guilty, overwhelmed, and helpless

Managing parenting while unwell led to a range of emotions including “guilt” (M01), “shame” (M03), being “overwhelm [ed]” (M13), “helplessness” (M06), and “sadness” (M12). Feelings of guilt and shame were frequently reported (*n* = 8) when participants felt they were not “do [ing] enough” (M01) or “my daughter was abandoned by me” (M12). This led to self-blame, with M06 blaming herself for her child’s addiction to video games, “If I were a normal mum, I would have cut off the internet” and “read books with him”. A few participants (*n* = 3) felt overwhelmed as “being a good mother” required “more physical effort, energy, and mental control” (M03) when unwell. Others (*n* = 3) felt self-doubt and helpless; “I doubt if I have the ability to do this [parenting]” (M13).

#### Self-acceptance

Although many participants (*n* = 12) experienced negative feelings associated with parenting, some (*n* = 5) tried to accept not being a “perfect” (M12) or an “ideal mother” (M11). One participant reflected:

I was possibly asking myself to be more perfect [as a mother]. I couldn’t accept that I wasn’t able to be extra good to her [the child] with all my heart and soul. So, [in this case,] I’d rather not be with her. This seems like a very extreme thought ... After seeing what I was asking of myself in this way, I have been trying to balance it as much as I can. (M12)

Similarly, another reported, “There’s no perfect mother and I have already done well enough” (M01).

### Perceptions about the impact of the mental illness on children

All participants except those with postnatal depression were concerned about the negative impact of their illness on children. Three sub-themes were identified: i) hereditary worries; ii) impact on children’s development; and iii) reducing the negative impact of mental illness on children.

#### Hereditary worries

Four participants expressed concern about the heritability of mental illness. M06 indicated that she was “so afraid that my child had inherited my illness” and M12 stated that “I felt guilty and scared ... a loss of hope that she might be perfect”. The hereditary fears extended to concerns about children’s future, e.g., “Will it affect her marriage and having children in the future because I have this illness?” (M13).

#### Impact on children’s development

One participant (who had OCD) described the impact of the illness on her son’s physical development (e.g., “poor body strength and coordination”) because she did not allow her son to crawl and touch “dirty floors” (M02). Others (*n* = 9) reported the impact of the illness on children’s psychological development in terms of being “very anxious” and “couldn’t focus in class” (M13), “timid and cautious” (M09), and “very poor in communication” (M06). Impacts varied depending on children’s ages. To illustrate, M12 reported that during her illness, her four-year-old daughter went from actively seeking out her mother’s company “Mum, read me a story” to “Mum, you can stay at home [to rest]. I’ll play with grandma” (M12). In contrast, another participant with a teenage child indicated that her harsh parenting led to “my child and I had been like strangers” (M10).

#### Reducing the negative impact of mental illness on children

Two participants described trying to protect their children from the illness by being “self-restrained” (M03), or “self-controlled” (M14). Another two distanced themselves from their children explaining that “Emotions are infectious. I was afraid of affecting my child” (M13) and “If I let her be around me, I might hurt her” (M12). A few participants (*n* = 2) drew on family support, for instance, M02 “left the child with the grandparents” who “would take the child to the park to tread water and play, just like any other child”. Others (n = 2) prepared their children for times of low moods (M03), reassuring the child that she was recovering (M13). In addition, M10 described “making up” with her son by positioning him as “the computer expert in our family”, “trust [ing]” and “respect [ing]” him. One participant reduced the “academic pressure” (M13) on her child, while another took her child for a psychological assessment (M01).

### Experiences of talking to children about mental illness

Six participants had previously discussed their illness with their children, explaining that they were “depressed” (M01), “have OCD” (M02), “have a mood illness” (M03), or “have a mental illness” (M06). One explained her bipolar disorder to her eight-year-old daughter by saying, “It’s a small problem and I may not be able to control myself. Mum is on medication now and then I’ll slowly calm down” (M03). Another participant of a 15-year-old son was cautious about the depth and detail of the explanations, not wanting to “scare” her child (M10). Three other participants explained their illness by saying that they were “tired” (M05), or “don’t sleep very well, so I don’t have a good temper” (M09).

A few participants (*n* = 2) believed that hiding the illness was ineffective because the child “knows that something is wrong with you” (M01). Some (*n* = 4) wanted to reassure their children as one explained when she lost her temper the child “would understand that it’s not her fault. … It’s solely because of my illness” (M03). Others (*n* = 3) described talking to children as a means of “reducing guilt” (M10) and as a rationale for a lack of discipline, “Your mother is a patient. I don’t have the energy to discipline you ... So, you better manage yourself” (M06).

Five participants did not discuss their illness to their children with the most commonly reported concern being the psychological burden on children; “She’ll worry about me and it may affect her studies” (M13). One participant thought it unnecessary for her children to know about her mental illness, “What else can they do if I let them know?” (M14). A few (*n* = 2) believed their children were “too young to understand” (M11) and others (*n* = 2) were not sure how to go about this, e.g., “I don’t know from which perspective, at what time, and how to talk about it” (M12). The difficulty of describing mental illness to children was stated by one participant of a four-year-old daughter: “If a child’s mother has a caesarean section, she’ll have a cut ... but mental illness is different. I can’t say, ‘There’s a little monster in mum’s head’” (M12).

### How having children impacts mothers’ mental illness and their recovery

In the first instance, having children motivated some participants (*n* = 6) to seek treatment with the hope of “bring [ing] back my child” from her relatives (M12), “not affect [ing] my child” (M09), and being able to “cook something nice for my daughter” (M13). A few participants (*n* = 3) managed negative and/or suicidal thoughts by focusing on caring for their children; “When I got triggered by something, I pulled myself back to cook for my son” (M06). Another reported having her children prevented her from thinking of suicide because “our children still need me” (M14).

All consistently reported the positive impact of children on their recovery. Being with children, seeing children’s growth and the “responsibility” (M13) of supporting children’s education provided some participants with “hope” (M11 & M13), with some comparing their children to “a little sun” who are “energetic and happy” (M12) and “good medicine” (M09). For example:

She [the daughter] is particularly keen to help me pick out clothes and wants me to look good. Her attitude has definitely had some good effects on me. (M14)

Moreover, one participant reported parenting provided her with opportunities to regain her previous abilities and skills. For example, she reported sending her child to another city for a competition “was a turning point in my whole recovery process ... I felt that my abilities were coming back, including the ability to communicate, to design travel itineraries, and to memorize” (M10). Simultaneously, others (*n* = 7) indicated that interactions with children could trigger or worsen their illness, e.g., by “becoming over-exhausted” (M14). Children’s poor academic performance was also triggering; “It made me anxious. ... [and] also worsened [my illness]” (M09).

### Support obtained and needed

In relation to support, three subthemes were identified: i) helpful support; ii) unhelpful support or a lack of support; and iii) support needed.

#### Helpful support

Two participants found it “helpful” (M12) to receive professional support for their parenting, with one realising after counselling that “it’s okay to be an 80-point mum when I’ m not well” (M12) and another reporting parenting courses as a way of “looking for strength” (M10). Two described being “lucky” (M09) and “relieved” (M10) for receiving sufficient support from their families in managing their finances, household chores, and parenting responsibilities. Friends were another source of support for a few mothers (*n* = 2); “I take my child, and my friend takes her child out … She plays with the two children, and I can rest for a while” (M01).

#### Unhelpful support or a lack of support

Overall, participants reported a lack of professional support for their parenting. Sometimes this was because they did not ask for it (*n* = 2), believing their illness was not serious, they did not have confidence in the service to provide parenting support, or there was no support to be found. M09 noted, “They [psychiatrists] really have an incredible amount of outpatient visits. They don’t have time to talk to you about such detailed things” (M09). Another participant received “conflicting” (M13) advice on pregnancy:

One psychiatrist said I could have a baby … but he didn’t say exactly why. Another psychiatrist … told me I couldn’t have a child as the illness was hereditary … The last one … didn’t advise on having or not having children. He didn’t take a stand. (M13)

Some (*n* = 6) described the ineffective support received from different family members including husbands who believed the participant was “overthinking” (M03), did not “do anything” and “just wanted me to do something good to the child” (M14), or “got hysterical and shouted at me, ‘What have you taught my son?!’” (M06). Several (*n* = 2) felt disappointed with their parents or parents-in-law for the lack of support even when “it’s the in-laws who should help with the childcare” (M11). Nonetheless, involving extended families for a few participants (*n* = 3) caused discipline problems with some grandparents being described as “liking to break boundaries” (M05), “spoiling” (M10), and not being able to supervise children’s academic work (M11). M14 explained what she considered to be the irreplaceable role of mothers despite the childcare support she received:

[*Sigh*] … They can’t help with anything other than everyday life ... like eating, dressing ... picking up and dropping off at school … But as for how well the child learns and how well the child grows, I still have to manage it myself. (M14)

Conflicting interactions with extended families burdened a few participants (*n* = 3), with M04 describing the “tense” co-caring relationship between her mother and mother-in-law leading to her “strained relationship” with her husband.

#### Support needed

Eleven participants reported wanting to talk about children to mental health professionals. M01 described wanting “emotional support” so that they would feel “not alone” and to know that their illness would “not necessarily have a negative impact on my child”. Some (*n* = 9) wanted parenting-related information**,** including hereditary issues involving mental illnesses (M13), methods of protecting children from the illness (M03), and ways to address children’s developmental challenges (M06).

One [of my questions to ask professionals] is whether the illness will be passed on to my child. … What should I do to prevent my child from getting the illness? ... If I had not had the child, I would ask, “Can I have a baby? If I want to have a baby, what should I do to prepare?” (M13)

Let’s say I get angry at my child ... how can I manage that with my child? Or is it okay if I don’t deal with it? If I need to deal with it, then what should I do? ... What do parents in our situation need to be aware of when we are with their children? (M03)

Psycho-education for the family including children, partners, and extended families was emphasized by three participants so that children can “understand that it’s not their fault that mum or dad has an emotional problem … [and] don’t blame themselves” (M03). For partners, one participant described “If only the professionals could convince my husband and tell him what my problem is” (M11). Other needs for professional support included addressing co-caring problems with parents and parents-in-law (M04).

Some (*n* = 8) highlighted a need for childcare when ill; “I need someone to care for my child. Once I had an acute episode where I really couldn’t care for my child … my son sitting on the sofa quite helplessly and watching the TV for I don’t know how long” (M06). A few (*n* = 2) wanted emotional support from family and friends, e.g., “When I wonder … if I’m affecting her [child], they can tell me … ‘Look, your daughter is [going] pretty good” (M01). Most participants (*n* = 11) thought it would be beneficial to talk to other parents with mental illness, so they would feel “not alone” (M01), and “less burdened” (M07), as well as “learn some lessons from other people” (M01). Concurrently, some (*n* = 5) noted that such peer groups might make them “more depressed” (M02), or “affect my illness” (M13) unless participants were “in treatment” (M13), had the same diagnosis (M02), or had recovered (M04 & M11).

## Discussion

The purpose of this study was to investigate the parenting experiences and needs of Chinese mothers with various mental health diagnoses. The value of motherhood and its positive impact on recovery was highlighted. Simultaneously, mothers described feeling guilty, sad, helpless, and overwhelmed. Significant issues embedded in the Chinese support systems remain to be addressed, including a lack of professional support for parenting, and unsupportive family relationships, often arising from an inadequate understanding of mental illness from husbands.

In this study, mothers saw motherhood as a central part of their identity that brought them “completeness” and a means of “contributing”. Similarly, other studies, involving Western mothers, have noted the importance of one’s mothering identity, and the sense of purpose and connectedness being a mother can provide, all key elements for recovery [32–34]. Moreover, the responsibility of motherhood and interactions with children motivated Chinese mothers to seek treatment and to “pull” themselves away from negative thoughts and symptoms, resonating again with studies of Western mothers [5, 12, 13, 35]. Conversely, the demands of childcare and some children’s poor academic performance left some mothers feeling exhausted or anxious, triggering or worsening their illness. These experiences align with studies conducted in China and Western countries which found that having children can impact mothers negatively, especially when disciplining children [13, 15]. Some research has found that compared to European-American mothers, Chinese-American mothers have high expectations and are willing to invest everything for their children’s education, due to the Confucian emphasis on education and respect for scholars [36, 37]. Therefore, it might be speculated that the impact of children’s academic performance on Chinese mothers with mental illness may differ from that of Western mothers. However, this speculation requires comparative research.

Consistent with studies in China and other countries [6, 15], mothers in this study described the stigma received from relatives. These mothers also experienced self-stigma and shame, especially when comparing themselves to “other parents”. Some were concerned that their children may suffer stigma because of their illness. Possibly because of public stigma, mothers in this study reported a lack of confidence in their parenting, and guilt about their children, consistent with other studies in the West and Japan [6, 7, 11, 13]. Reupert et al. [38] argued that the (in) ability of mothers to fulfil the socially standardized gender role and idealized parenting role underpins the stigmatization associated with being a mother with mental illness. The pervasive nature of stigma, described by the mothers in this study, poses a threat to their recovery and help-seeking [38, 39]. It also may stop them from talking to children about their illness, as others have found [40, 41].

Chinese mothers reported warm and nurturing parenting strategies when feeling well, and parenting challenges when unwell. Likewise, mothers in the US and Australia experienced good and bad days [14], and different family interactions (including parenting) at well and unwell times [35]. In this study, when mothers were feeling unwell some shut down and did not communicate with their children; some lost their temper with their teenagers especially when facing discipline challenges. One mother with psychosis in the current study described certain worrying parenting behaviours (e.g., hitting or harming children causing by psychosis), highlighting a critical need to address such behaviours in some families.

The mothers in this study employed several strategies that they believed would protect their children from their mental illness, in terms of “self-control” and distancing themselves from their children. Similarly, Chinese mothers in Chan, Ho [15] study described employing “emotional control”, seeking strategies to manage their anger such as walking away and ignoring the child. It is unclear whether these strategies are effective nor whether such behaviours are specific to Chinese mothers. However, Montgomery, Tompkins [42] suggested “strategies of pretences” (p. 27) can reduce mothers’ presence and make mental illness invisible for their children, costing some mothers their “integrity” (p. 24).

Talking to children about their mental illness was another method some mothers employed to protect their children. Others identified various barriers to talking to children, such as the fear of burdening children, and not having (or not perceiving to have) the skills to talk about their illness with children. Similar concerns have been raised by mothers in the U.K. and Japan [39, 40]. Nonetheless, it is important for children to know about their parents’ illness, as without this knowledge they may feel that their parents’ illness is their fault [43, 44]. Falkov [40] argued that family conversations about parental mental illness can benefit both parents and children. Interventions that promote family conversations, e.g., “Let’s talk about children” [45], might be useful, once adapted to the Chinese context. The need to talk about children with mental health professionals was highlighted and is a training implication arising from this study.

In contrast to studies from western countries [6], but similar to those from Japan [39] and China [15, 46], Chinese mothers in this study were not concerned about, nor did they report losing custody of their children due to their mental illness. Unlike the legal system in some US states, which removes children from parents on the grounds of mental illness [47], child protection policies in China are relatively non-interventionist [48]. Moreover, under a Confucian perspective emphasizing family and parental responsibilities [49], the broad family system plays an important role in supporting parents with a mental illness informally and this too may have negated the need for the involvement of child protection agencies. Additionally, although the mental health system in China is reforming, mental health resources (e.g., hospital beds, psychoeducation packages) and services are overall lower than in high-income countries [50]. Embedding parenting into mental health service delivery is challenging in many Anglophile countries [51, 52] so it is perhaps not surprising that the Chinese mothers in this study described receiving limited professional support for their parenting and relying mostly on support from their families, even though at times this was unhelpful.

Mothers who received sufficient support from their families and friends considered themselves “lucky”, suggesting that such support was not always available. Unsupportive husbands appeared to lack an understanding of mental illness, did not engage in parenting, and blamed mothers for children’s problems. This would suggest that unsupportive husbands perform a traditionally Chinese male role, believing that they are responsible for earning money while childcare and household chores are women’s responsibilities [19].

In this study, parents and parents-in-law were described as a source of economic and practical support, in contrast to Western mothers who tended to seek support from their partners and their own mothers [12, 13]. Parents-in-law in this study played an important role in supporting Chinese mothers. However, the co-caring relationship between the mothers and their in-laws was at times conflictual, sometimes leading to tensions between the mother and her husband. Family conflicts are common in Chinese families when extended families provide childcare support [22, 53], and conflicts with mother-in-law are a predictor of PPD [54, 55]. Therefore, the need for resolving conflictual co-caring relationships is necessary, especially for mothers who have PPD.

Mothers also indicated a need for childcare support, especially during an acute episode or hospitalisation, a common need identified across different cultural and societal contexts [5, 15, 56]. The need for emotional support, including an understanding of their illness and reassurance that their children were doing well, might be facilitated through family psychoeducation. Some mothers also welcomed peer support groups, with some wanting to be matched with others with the same diagnosis or recovery stage.

### Limitations and future research

In this exploratory study, participants with various mental health diagnoses were purposely included, and their diagnoses were self-reported. Van Santvoort, Hosman [4] found that the impact on family life and children may vary depending on the parents’ diagnoses. It is also noteworthy that different mental illness symptoms, severity, and chronicity may impact mothers’ parenting in various ways [57, 58], though this was not the focus of this study. Future studies might compare the experiences of Chinese mothers with different formal diagnoses and varying levels of functioning and symptomatology. In addition, participants were mostly well-educated, with a full-time job and above-average household income. As mothers with mental illness are more likely to experience socio-economic difficulties than those without mental illness [59], future research might include Chinese mothers with different levels of education, employment and economic status. Likewise, the ages of participants’ children were broad. Given that children’s age can moderate the impact of mental illness on parenting [60], research is required to investigate the experiences of Chinese mothers whose children are at a certain developmental stage. The lack of triangulation with other informants is another possible limitation [61], especially considering that the perspectives of children, partners, alternative caregivers of children, and mental health professionals can differ [46, 62].

### Implications

Depending on their workplace, mental health professionals might need training in how to address clients’ common concerns about parenting, and their hereditary worries involving mental illness. Anti-stigma campaigns might be developed to address both public and self-stigma [38]. Such campaigns could include psychoeducational resources, and where mothers might be supported to talk to their children and other family members about their mental illness. Additionally, family-focused interventions might be developed to address conflicting co-caring relationships within the family, especially between mothers and their in-laws. Finally, peer-support groups for mothers could be developed.

## Conclusions

This study demonstrated the parenting experiences and needs of Chinese mothers with mental illness. Despite vastly different socio-cultural contexts and mental health services, the study identifies important similarities with many western mothers with mental illness. However, the lack of professional support meant Chinese mothers had to rely on their families which brought both benefits and burdens.

## Supplementary Information

**Additional file 1.** Interview schedule.

## Data Availability

The datasets generated and/or analysed during the current study are not publicly available due to the sensitive nature of narratives shared that may possibly disclose participants’ identities, but are available from the corresponding author on reasonable request.

## Abbreviations

IPA: Interpretative phenomenological analysis
PPD: Postpartum depression
OCD: Obsessive-compulsive disorder

## References

[1] Leijdesdorff S, van Doesum K, Popma A, Klaassen R, van Amelsvoort T (2017). Prevalence of psychopathology in children of parents with mental illness and/or addiction: an up to date narrative review. Curr Opin Psychiatry.

[2] Hser YI, Lanza HI, Li L, Kahn E, Evans E, Schulte M (2015). Maternal mental health and children's internalizing and externalizing behaviors: beyond maternal substance use disorders. J Child Fam Stud.

[3] Devlin JM, O’Brien LM (1999). Children of parents with mental illness. I: an overview from a nursing perspective. Aust N Z J Ment Health Nurs.

[4] van Santvoort F, Hosman CM, Janssens JM, van Doesum KT, Reupert A, van Loon LM (2015). The impact of various parental mental disorders on children's diagnoses: a systematic review. Clin Child Fam Psychol Rev.

[5] Diaz-Caneja A, Johnson S (2004). The views and experiences of severely mentally ill mothers. Soc Psychiatry Psychiatr Epidemiol.

[6] Dolman C, Jones I, Howard L (2013). Pre-conception to parenting: a systematic review and meta-synthesis of the qualitative literature on motherhood for women with severe mental illness. Arch Womens Mental Health.

[7] Perera DN, Short L, Fernbacher S (2014). There is a lot to it: being a mother and living with a mental illness. Adv Ment Health.

[8] Mowbray CT, Oyserman D, Ross S (1995). Parenting and the significance of children for women with a serious mental illness. J Mental Health Adm.

[9] Mizock L, Merg AL, Boyle EJ, Kompaniez-Dunigan E (2018). Motherhood reimagined: experiences of women with SMI surrounding parenting. Psychiatr Rehabil J.

[10] Reupert A, Price-Robertson R, Maybery D (2017). Parenting as a focus of recovery: a systematic review of current practice. Psychiatr Rehabil J.

[11] Ueno R, Kamibeppu K (2008). Narratives by Japanese mothers with chronic mental illness in the Tokyo metropolitan area: their feelings toward their children and perceptions of their children's feelings. J Nerv Ment Dis.

[12] van der Ende PC, van Busschbach JT, Nicholson J, Korevaar EL, Van Weeghel J (2016). Strategies for parenting by mothers and fathers with a mental illness. J Psychiatr Ment Health Nurs.

[13] Ackerson BJ (2003). Coping with the dual demands of severe mental illness and parenting: the parents' perspective. Fam Soc.

[14] Venkataraman M, Ackerson BJ (2008). Parenting among mothers with bipolar disorder: strengths, challenges, and service needs. J Fam Soc Work.

[15] Chan SYY, Ho GWK, Bressington D (2019). Experiences of self-stigmatization and parenting in Chinese mothers with severe mental illness. Int J Ment Health Nurs.

[16] van der Ende PC, van Busschbach JT, Nicholson J, Korevaar EL, van Weeghel J (2014). Parenting and psychiatric rehabilitation: can parents with severe mental illness benefit from a new approach?. Psychiatr Rehabil J.

[17] Boursnell M (2012). Assessing the capacity of parents with mental illness: parents with mental illness and risk. Int Soc Work.

[18] Källquist A, Salzmann-Erikson M (2019). Experiences of having a parent with serious mental illness: an interpretive meta-synthesis of qualitative literature. J Child Fam Stud.

[19] Chen L, Reupert A, Vivekananda K (2021). Chinese mothers' experiences of family life when they have a mental illness: a qualitative systematic review. Int J Ment Health Nurs.

[20] Shek DT, Sun RC, Selin H (2014). Parenting in Hong Kong: Traditional Chinese cultural roots and contemporary phenomena. Parenting across cultures: Childrearing, motherhood and fatherhood in non-Western cultures.

[21] Park M, Chesla C (2007). Revisiting Confucianism as a conceptual framework for Asian family study. J Fam Nurs.

[22] Goh ECL (2011). China's one-child policy and multiple caregiving: raising little suns in Xiamen: Routledge.

[23] Leung S, Arthur D, Martinson I (2005). Stress in women with postpartum depression: a phenomenological study. J Adv Nurs.

[24] Gao L, Chan SW, You L, Li X (2010). Experiences of postpartum depression among first-time mothers in mainland China. J Adv Nurs.

[25] Chen-Bouck L, Patterson MM, Chen J (2019). Relations of collectivism socialization goals and training beliefs to Chinese parenting. J Cross-Cult Psychol.

[26] Wu DY, Tseng WS. Introduction: The characteristics of Chinese culture: Chinese culture and mental health: Academic Press; 1985. p. 3–13. 10.1016/B978-0-12-701630-6.50007-1.

[27] Ding G, Yu J, Vinturache A, Gu H, Lu M (2018). Therapeutic effects of the traditional “doing the month” practices on postpartum depression in China. Am J Psychiatr.

[28] Yue A, Gao J, Yang M, Swinnen L, Medina A, Rozelle S (2018). Caregiver depression and early child development: a mixed-methods study from rural China. Front Psychol.

[29] Smith JA, Flowers P, Larkin M (2012). Interpretative phenomenological analysis: SAGE.

[30] Smith JA (2011). Evaluating the contribution of interpretative phenomenological analysis. Health Psychol Rev.

[31] Anney VN (2014). Ensuring the quality of the findings of qualitative research: looking at trustworthiness criteria. J Emerging Trends Educ Res Policy Stud.

[32] Hunt MG, Stein CH (2012). Valued social roles and measuring mental health recovery: examining the structure of the tapestry. Psychiat Rehabil J.

[33] Leamy M, Bird V, Le Boutillier C, Williams J, Slade M (2011). Conceptual framework for personal recovery in mental health: systematic review and narrative synthesis. Br J Psychiatry.

[34] Reupert A, Maybery D, Cox M, Scott SE (2015). Place of family in recovery models for those with a mental illness. Int J Ment Health Nurs.

[35] Marston N, Maybery D, Reupert A (2016). Parent perceptions comparing times of parental mental wellness and illness using a family functioning model. J Fam Stud.

[36] Huang GH, Gove M (2015). Asian parenting styles and academic achievement: views from eastern and western perspectives. Education..

[37] Chao RK (1996). Chinese and European American mothers' beliefs about the role of parenting in children's school success. J Cross-Cult Psychol.

[38] Reupert A, Gladstone B, Helena Hine R, Yates S, McGaw V, Charles G, Drost L, Foster K (2021). Stigma in relation to families living with parental mental illness: an integrative review. Int J Ment Health Nurs.

[39] Ueno R, Kamibeppu K (2012). Perspectives of Japanese mothers with severe mental illness regarding the disclosure of their mental health status to their children. Arch Psychiatr Nurs.

[40] Falkov A, Cowling V (2004). Talking with children whose parents experience mental illness. Children of parents with mental illness 2: personal and clinical perspectives.

[41] Ballal D, Navaneetham J (2018). Talking to children about parental mental illness: the experiences of well parents. Int J Soc Psychiatry.

[42] Montgomery P, Tompkins C, Forchuk C, French S (2006). Keeping close: mothering with serious mental illness. J Adv Nurs.

[43] Riebschleger J (2004). Good days and bad days: the experiences of children of a parent with a psychiatric disability. Psychiatr Rehabil J.

[44] Gladstone BM, Boydell KM, Seeman MV, McKeever PD (2011). Children’s experiences of parental mental illness: a literature review. Early Intervent Psychiatry.

[45] Cooper V, Reupert A (2016). “Let’s talk about children” resource: a parallel mixed method evaluation. Soc Work Ment Health.

[46] Xue M (2020). Understanding the experience of living with a parent with severe mental illness in China from multiple perspectives: a qualitative exploratory study: Harvard Medical School.

[47] Kaplan K, Kottsieper P, Scott J, Salzer M, Solomon P (2009). Adoption and safe families act state statutes regarding parents with mental illnesses: a review and targeted intervention. Psychiatr Rehabil J.

[48] Chui CH-K, Jordan L (2018). Child protection in China: threats and opportunities. Asia Pac J Soc Work Dev.

[49] Zhao F, Hämäläinen JEA, Chen HL (2017). Child protection in China: changing policies and reactions from the field of social work. Int J Soc Welf.

[50] Que J, Lu L, Shi L. Development and challenges of mental health in China. Gen Psychiatry. 2019;32(1). 10.1136/gpsych-2019-100053. 10.1136/gpsych-2019-100053 PMC6551437 31179426

[51] Allchin B, Goodyear M, O'Hanlon B, Weimand BM (2020). Leadership perspectives on key elements influencing implementing a family-focused intervention in mental health services. J Psychiatr Ment Health Nurs.

[52] Maybery D, Reupert A (2009). Parental mental illness: a review of barriers and issues for working with families and children. J Psychiatr Ment Health Nurs.

[53] Settles BH, Sheng X, Zang Y, Zhao J, Chan K (2013). The one-child policy and its impact on Chinese families. International handbook of Chinese families.

[54] Mao Q, Zhu LX, Su XY (2011). A comparison of postnatal depression and related factors between Chinese new mothers and fathers. J Clin Nurs.

[55] Lee D, Yip A, Leung T, Chung T (2004). Ethnoepidemiology of postnatal depression: prospective multivariate study of sociocultural risk factors in a Chinese population in Hong Kong. Br J Psychiatry.

[56] Rampou AM, Havenga Y, Madumo M (2015). Parenting experiences of mothers living with a chronic mental illness. Health SA Gesondheid.

[57] Seeman MV, Reupert A, Maybery D, Nicholson J, Göpfert M, Seeman MV (2015). Schizophrenia and motherhood. Parental psychiatric disorder: Distressed parents and their families.

[58] Oyserman D, Mowbray CT, Meares PA, Firminger KB (2000). Parenting among mothers with a serious mental illness. Am J Orthop.

[59] Luciano A, Nicholson J, Meara E (2014). The economic status of parents with serious mental illness in the United States. Psychiatr Rehabil J.

[60] Lovejoy MC, Graczyk PA, O'Hare E, Neuman G (2000). Maternal depression and parenting behavior: a meta-analytic review. Clin Psychol Rev.

[61] Rothbauer P, LMT G (2008). Triangulation. The Sage encyclopedia of qualitative research methods: Sage publications.

[62] Reupert A, Maybery D (2007). Families affected by parental mental illness: a multiperspective account of issues and interventions. Am J Orthop.

