# Supplementary material for: Parenting experiences of Chinese mothers living with a mental illness
Source: BMC Psychiatry. 2021 Nov 23;21:589. doi: 10.1186/s12888-021-03581-9 (PMC8609737; doi:10.1186/s12888-021-03581-9)
Supplement: Supplementary file 1 — Additional file 1. Interview schedule. [file 12888_2021_3581_MOESM1_ESM.docx]

**Interview schedule**

# Section A: Parenting experience

1. Everyone may have thought of an ideal family. From your perspective, what is a mother like in the ideal family? What is a father like?

*Possible prompts: For example, what should a mother/father do in the family? What is she/he responsible for? How does she/he care and educate the children? How does she/he cooperate with other families?*

*Note: Asking parents both mothers’ and fathers’ roles can help to understand their parenting and co-parenting practice in families because the illness may influence their partners’ role as a parent as well.*

1. How might your illness impact your role as a father/mother, if at all?

*Possible prompts: Do you feel that there are some things you can’t do as a parent because you have a mental illness? Why or why not?*

1. Parenting can bring both happiness or sadness. Which kind of emotion do you experience more, happiness or sadness?

(1) Tell me about the joy that parenting provides for you, if at all?

(2) Tell me about the sadness that parenting provides for you, if at all?

*Possible prompts: For example? What makes you happy/ upset?*

1. **[Check before asking]** Have you ever thought of comparing with other parents? For example, the ones who don’t have a mental illness?

(1) How do you feel your participation in parenting compares to others who do not have a mental illness?

(2) How do you feel when you compare yourself with others? How do you deal with the feeling?

*Note: The question may elicit upset, two steps can be undertaken here:*

*a. Early warning before asking: the following question may be a little sensitive, if you feel upset after I ask, please tell me, we can skip that question if you want.*

*b. Check the participant’s state before asking: if the participant is in good condition in the previous questions, then ask; if she/he is upset in answering previous questions, then skip.*

1. Some people may have experienced this, while some may not. Have you ever been discriminated against or felt prejudiced by others because you are a parent with a mental illness?

*Now we come to the second topic, parenting and illness or recovery.*

# Section B: Parenting and illness/recovery

1. Tell me the differences when you are well and ill. *(Possible prompts: What is your typical day like when you are well/ill?)*

(1) What do you do when you feel well? What do your children do? How about other families?

(2) What do you do when you feel ill? What do your children do? How about other families?

(3) How is that different for you as a parent?

*Possible prompts: For example, how is that different when you are talking with your children, playing with them, caring for them, or educating them?)*

(4) Can the children feel the difference in you? How do you know that?

*Possible prompts: How do you feel when you know the children can feel the difference?*

1. How might having children impact your illness, if at all?

*Possible prompts: Including the birth of children, the characteristics of children, and the process of parenting...*

1. Tell us about how having children influences your recovery?

(1) Recovery has two meanings. The first meaning is relatively easy to understand. It refers to physical recovery, which can usually be achieved by taking medicine or psychological treatment. For example, if we have a cold, our physical recovery means fever reduction, no cough, and no runny nose. When we talk about mental illness, it's actually like we have a cold in spirit, and recovery is the reduction of symptoms, such as insomnia, depression or anxiety, and hallucinations. In short, physical recovery means a decrease in illness and moving in a better direction.

**How might having children influence you to pursue physical recovery, if at all?**

*Possible prompts: What are the positive impacts? How about negative impacts? For example, is there any impact on taking medicine or seeking treatment?*

(2) The second meaning of recovery is psychological recovery. For example, some of us may have chronic diseases, such as gastritis, which may occasionally occur and cause stomach pain and discomfort, but it does not affect our attitude of living a meaningful life. The same is true in mental illness. Although we still have some symptoms, such as emotional fluctuations or occasional physical conditions, we still believe that life is meaningful and willing to live with these symptoms with a positive attitude. In short, psychological recovery means hopefulness for life, although the illness has not been healed yet.

**How might having children influence you to pursue psychological recovery, if at all?**

*Possible prompts: What are the positive impacts? How about negative impacts?)*

1. Has the pandemic had any impact on your illness, parenting, and your family relationships? If so, what kind of impact is it?

*Possible prompts: How have the precautionary measures affected you, such as home quarantine/school suspension/changes in work practices/changes in hygiene requirements/uncertain future?*

1. **[Early warning before asking]** For those who do not live with children/ have lost custody:

(1) In our last confirmation call, you mentioned that you are not living with your children. Can you tell me more about this?

*Possible prompts: When? In what ways? Why did you not live with your child/ren? How did you feel? How did you cope?*

(2) (If the reason for not living together with children is that parents lost custody) Tell me about the experience of losing custody of your children?

*Possible prompts: When? In what ways? Why did you lose custody? How did you feel? How did you cope?*

(2) How did that impact your illness?

*Possible prompts: What is the positive impact? How about the negative impact?*

*Now we come to the third topic, which relates to your parental role and children*

# Section C: The importance of parental role

1. Everyone plays different roles in life. For example, we are sons or daughters of our parents, we are husband, wife or partner of someone, we are an employee or a leader in our working field… Every role contains both responsibilities and obligations we have to fulfil and the rights we can enjoy. The importance of these roles for each of us must be different.

**How important is your role as a parent, if it is?**
(1) If the answer is “important”, ask: What are the important influences of being a parent on you? Can you give me an example? How would your life be different without becoming a parent?
(2) If the answer is “not important”, ask: What do you think is more important to you than being a parent? What is the difference between this and parenting?
(3) If the participant says “don’t know”, ask: Do you say you don’t know because you don’t know how to evaluate the extent of importance? If so, can you tell me how parenting has affected your life or life?

1. How important are your children to you?

(1) If the answer is “important”, ask: What important impact does your child have on you? Can you give me an example? What would be different for you without children?

(2) If the answer is “not important”, ask: What do you think is more important to you than the children? why?

(3) If the participant says “don’t know”, ask: Do you say you don’t know because you don’t know how to evaluate the extent of importance? If so, can you tell me what influence your child has on your life?

*Now we come to the fourth topic, which focuses on the support you obtained and need*

# Section D: Support system

1. What do other family members do when you are unwell?

*Possible prompts: Who do you refer to when you talk about family members? What would they say/do?)*

1. Who do you call on for help, if anyone? Probe for family, friends, professionals?

(1) What kind of problems would you usually ask them for help with? How would they help you? How do you feel about their help?

*Possible prompts: professionals refer to social workers, counsellors, and clinic doctors.*

(2) Was their help helpful for you? What was the helpful part? What was the unhelpful part which may even make you feel worse?

1. What help do you need as a parent with a mental illness? *(Possible prompts: what and how?)*
2. How can your family or friends help you in your role as a parent with a mental illness?

*Possible prompts: explore different family members – partner, their parent, parent-in-law, and other relatives or friends.*

1. What more could be done by mental health professionals, if anything?
2. When you go for treatment, does anyone ever talk to you about your children?

(1) If someone does, who are they? What did you talk about?

*Possible prompts: For example, when you go to a psychiatric clinic, or when you go to see a counsellor, does your doctor or counsellor talk about your children with you?*

(2) Do you want them to? Why or why not?

1. What kind of online support do you need as a parent with a mental illness, if any? What sort of information would this include?
2. Would it be useful to talk to other parents with a mental illness? Why or why not?
3. Have you ever talked to your children about your illness? Why or why not?

Do you think it would be important for them to know? Why or why not?

What might stop you from talking to your children about your illness?

If not you, who else might be asked to talk to your children about your illness?

*Now it is the last topic associated with your vision for the future*

# Section E: Hope for future]

1. What hopes do you have for the future? For you? For your children? For your family?
